# Preliminary field evaluation and exploratory external-association analysis of a modified wellness questionnaire for short-term monitoring in elite male water polo players

**DOI:** 10.3389/fspor.2026.1902609

**Published:** 2026-08-18

**Authors:** Zsolt Varga, Gergely Lukács, András Gárdonyi, Zoltán Tarjányi, Nándor Prikidánovics, Kinga Shenker-Horváth, Gábor Pavlik, Gábor Géczi, Sándor Sáfár, Krisztián Havanecz

**Affiliations:** 1 Hungarian Water Polo Federation, Budapest, Hungary; 2 Physical Education and Exercise Center, Medical School, University of Pécs, Pécs, Hungary; 3 Heart and Vascular Centre, Semmelweis University, Budapest, Hungary; 4 Center for Sports Nutrition Science, Hungarian University of Sports Science, Budapest, Hungary; 5 Department of Health Sciences and Sport Medicine, Hungarian University of Sports Science, Budapest, Hungary; 6 Department of Sport Management, Hungarian University of Sports Science, Budapest, Hungary; 7 Training Theory and Methodology Research Center, Hungarian University of Sports Science, Budapest, Hungary

**Keywords:** athlete monitoring, recovery, water polo, wellness, wellness questionnaire

## Abstract

**Introduction:**

Monitoring athletes' perceived wellness has become an important component of daily tasks of practitioners, yet the properties of brief wellness questionnaires remain insufficiently examined in specific team sport contexts. The purpose of this study was to describe the internal consistency, between-athlete reliability, and estimates of measurement error for a modified wellness questionnaire during short-term field monitoring and to explore associations with selected external monitoring variables.

**Methods:**

Routinely collected data from eighteen elite male water polo players were retrospectively analyzed across 12 repeated measurement occasions, resulting in 216 questionnaire observations. The questionnaire assessed sleep quality, physical and, upper- and lower-body fatigue, mental fatigue, and stress, from which composite recovery and fatigue scores were derived. All items were scored so that higher values indicated a more favorable status. External variables included sleep duration, heart rate variability (HRV), and daily summed session-rating of perceived exertion (s-RPE); additional lagged models examined potential delayed associations with recovery and fatigue.

**Results:**

Most wellness indicators demonstrated low between-athlete reliability, particularly sleep quality and recovery-related items, indicating substantial within-athlete variability over time. Internal consistency was acceptable for the recovery composite and good for the fatigue composite. SEM values were 0.61 for recovery and 0.59 for fatigue, with corresponding MDC_95_ values of 1.69 and 1.64. Within-questionnaire mixed-effects models showed that recovery was associated with the stress score across measurement occasions. Exploratory external-association analyses showed that sleep duration was associated with perceived sleep quality and recovery, while HRV was associated with the stress score. Daily summed s-RPE was not meaningfully associated with concurrent wellness scores, although higher previous day s-RPE was associated with less favorable next-day fatigue.

**Conclusion:**

The modified wellness questionnaire showed interpretable internal score patterns and selected exploratory external associations during short-term descriptive monitoring in this specific elite water polo setting. However, the findings do not establish full psychometric validation, responsiveness, or suitability for training prescription decisions.

## 1 Introduction

Athlete wellness has become a central construct in contemporary sport science and sport psychology, particularly within elite performance environments where physical, psychological, and social demands interact dynamically. Perceived wellness reflects athletes' subjective evaluations of their physical and psychological states and has been shown to provide meaningful insight into training readiness, fatigue, recovery status, and overall functioning (1, 2). In high-performance sport, regular monitoring of perceived wellness is widely used to inform training decisions, support recovery strategies, and reduce injury risk (3).

Wellness and wellbeing are multidimensional constructs that encompass physical, psychological, and social components of human functioning. Conceptual models of athlete wellness emphasize the interaction between subjective experiences (e.g., mood, vitality, perceived health) and functional outcomes such as performance and adaptation (4, 5). Within sport psychology, perceived wellness is typically understood as a subjective indicator reflecting athletes' integrated appraisal of their physical readiness, psychological state, and recovery status (6). Such subjective evaluations are particularly valuable in elite sport contexts, where internal states may fluctuate rapidly in response to training load (7), competition stress, and recovery demands.

Subjective monitoring tools, including wellness questionnaires, have gained widespread acceptance in elite sport due to their sensitivity to short-term changes in athlete readiness and fatigue (8). From a monitoring perspective, such tools are relevant because they can capture state-like fluctuations within athletes over time, rather than only stable between-athlete differences. Compared to objective measures alone, subjective indicators often demonstrate stronger associations with rating of perceived exertion, recovery quality, and subsequent performance outcomes (6, 9). Importantly, athlete-reported wellness measures provide context-specific information that may not be captured by physiological or external training load metrics, reinforcing their value as complementary tools within integrated monitoring systems (10).

In applied elite sport environments, the feasibility of measurement tools is a critical consideration. Brief questionnaires are often preferred due to time constraints, athlete compliance, and the need for repeated assessments across training cycles. From a psychometric perspective, shorter scales may raise concerns regarding reliability and construct coverage. However, the methodological literature acknowledges that well-designed brief instruments can demonstrate acceptable reliability and validity when their purpose is clearly defined (11, 12). In the context of athlete monitoring, the primary aim is not clinical diagnosis but the efficient detection of meaningful changes in perceived status, supporting the use of concise, pragmatically oriented measures (13).

The importance of sport-specific measurement has been increasingly emphasized in sport psychology research, particularly in elite contexts where generic instruments may lack ecological validity. Sport-specific adaptations of psychological measures have been shown to enhance sensitivity and practical relevance by aligning assessment content with the unique demands of a given sport (14). In water polo, the combination of high-intensity intermittent activity, physical contact and tactical complexity underscores the need for assessment tools that are both contextually relevant and practically implementable within daily training environments.

Water polo is a physically demanding intermittent team sport characterized by repeated high-intensity swimming efforts, frequent physical contact, and substantial cognitive and emotional demands. Elite water polo players are often exposed to congested competition schedules and high training loads, which may adversely affect both physical and psychological aspects of wellness (15). Consequently, practical tools capable of efficiently monitoring perceived wellness are particularly relevant in water polo, where rapid fluctuations in readiness and fatigue are common.

Several questionnaires have been developed to assess athlete wellness and related constructs, such as fatigue, recovery, and mood states (1, 16–18). Brief and single-item self-report measures are also used in team sport monitoring contexts, although their interpretation requires attention to measurement quality and practical purpose (19). When an existing monitoring tool is modified for a sport-specific context, evidence is needed not only for internal consistency, but also for content relevance, scoring transparency, and the interpretation of short-term score changes within the target population. In applied settings, coaches and sport practitioners often prefer brief, easy-to-administer tools that can be integrated into daily monitoring routines without increasing athlete burden (20). Importantly, modifications or abbreviations of existing questionnaires may compromise their psychometric properties, underscoring the need for systematic validation when instruments are adapted for specific sports or populations.

To address these practical constraints, a modified wellness questionnaire was developed to capture key dimensions of perceived wellness relevant to elite water polo players. The modification process aimed to balance theoretical coverage of the wellness construct with the practical demands of daily athlete monitoring. Given that changes to questionnaire structure, item composition, or scoring procedures may alter measurement properties, modified instruments should be evaluated stepwise within their intended context of use. Such early evaluations can clarify score behavior, feasibility, and preliminary internal measurement characteristics before broader validation studies are conducted.

In measurement-property research, the evidential value of a study depends on the specific measurement question being addressed. For athlete monitoring instruments, an initial field-based evaluation can provide useful information on how scores behave during repeated use in the intended applied context, even when it does not establish full psychometric validation. Accordingly, the primary objective was to describe the internal consistency, between-athlete reliability, and estimates of measurement error for the modified wellness questionnaire during short-term repeated use in its intended setting. The secondary objective was to explore associations between questionnaire scores and selected external monitoring variables.

## 2 Materials and methods

### 2.1 Study design

This retrospective observational study used routinely collected monitoring data from 12 measurement occasions during a two-week national team training camp between 8 and 22 December 2025. Questionnaire data were collected repeatedly from each player throughout the monitoring period. Questionnaire scores were linked to routine sleep, HRV, and training load records collected during the same monitoring period. No intervention or group allocation was involved.

### 2.2 Participants

Participants were selected from the Hungarian national team training camp roster, with both field players and goalkeepers eligible for inclusion. Eligibility for the analysis required participation throughout the full two-week monitoring period. Although the roster expanded to 28 players in the second week of the training camp, the additional 10 players joined after completing their club competition commitments and did not participate throughout the full monitoring period. Data were collected from these players, but their observations were not included in the analysis. Accordingly, the analytical sample comprised eighteen (*N* = 18) elite male water polo players (age: 25.2 ± 4.2 years, height: 193.6 ± 5.2 cm, weight: 96.9 ± 7.0 kg, all data are presented as mean ± SD). The athletes represented different professional clubs and were actively competing at the senior international elite level during the data collection period. All participants provided written informed consent for the research use of their monitoring data. The study was conducted in accordance with the Declaration of Helsinki, and the protocol for the retrospective analysis was approved by the local institutional ethics committee (MTSE-KEB/No10/2026, 22 April 2026).

### 2.3 Data collection circumstances

Data were collected during a national team training camp. The camp was more of an intensive preparation period than the usual club training environment. All athletes were based at the same training location and followed the same daily training schedule. During the study period, players trained with the national team and not with their respective clubs.

Athletes were not experimentally isolated from their usual social environment, and the use of personal communication devices or social media was not restricted by the study protocol. Routine medical and sport science support was available throughout the monitoring period when required; however, no additional psychological intervention was introduced as part of the study. These details are reported to document the applied data collection context and to support reproducibility of the monitoring procedure.

### 2.4 Training program

During the data collection period, players followed a structured training program in the national team consisting of dry-land strength and in-water sessions. Each training day typically included a ∼60-minute strength and conditioning session, followed by a ∼120-minute water-based training session.

Strength training sessions consisted of mobility and neuromuscular activation exercises, followed by circuit-based training targeting strength-speed, core stability, and explosive exercises. In-water sessions included swimming-based warm-up activities, ball-handling drills, and game-based situations (e.g., 6 vs 6 play), performed in single-goal or double-goal formats under varying tactical conditions including cognitive tasks.

### 2.5 Modified wellness questionnaire

The modified wellness questionnaire was developed for applied daily monitoring in elite water polo, drawing on the original wellness questionnaire framework described by Hooper and Mackinnon (1995) (1). Item selection was guided by commonly used athlete wellness domains reported in the literature, with particular emphasis on perceived sleep quality, recovery, fatigue, and stress. The final version was adapted to the practical needs of the national team monitoring environment to ensure brevity and feasibility for repeated administration in an elite sport setting. Before implementation, the modified item set was reviewed by two members of the coaching and sport-science staff with experience in elite water polo to assess item relevance, clarity, and practical feasibility for daily use. The pragmatic adaptation process involved several steps. First, the domains of sleep quality, recovery, fatigue, and stress were selected based on commonly used wellness monitoring frameworks and their relevance to elite water polo. Second, item wording was reviewed by the members of the coaching and sport science staff to ensure clarity, sport-specific relevance, and feasibility for repeated administration during the training camp. Third, the final item set was kept deliberately brief to minimize athlete burden and support daily use in the national team environment. The goal of this process was pragmatic adaptation to the daily monitoring environment rather than a fully redeveloped instrument. The final item set focused on sleep quality, recovery, fatigue and stress and was kept deliberately brief to support daily use; mood, appetite, and muscle soreness were not assessed as separate domains.

**Figure 1:**
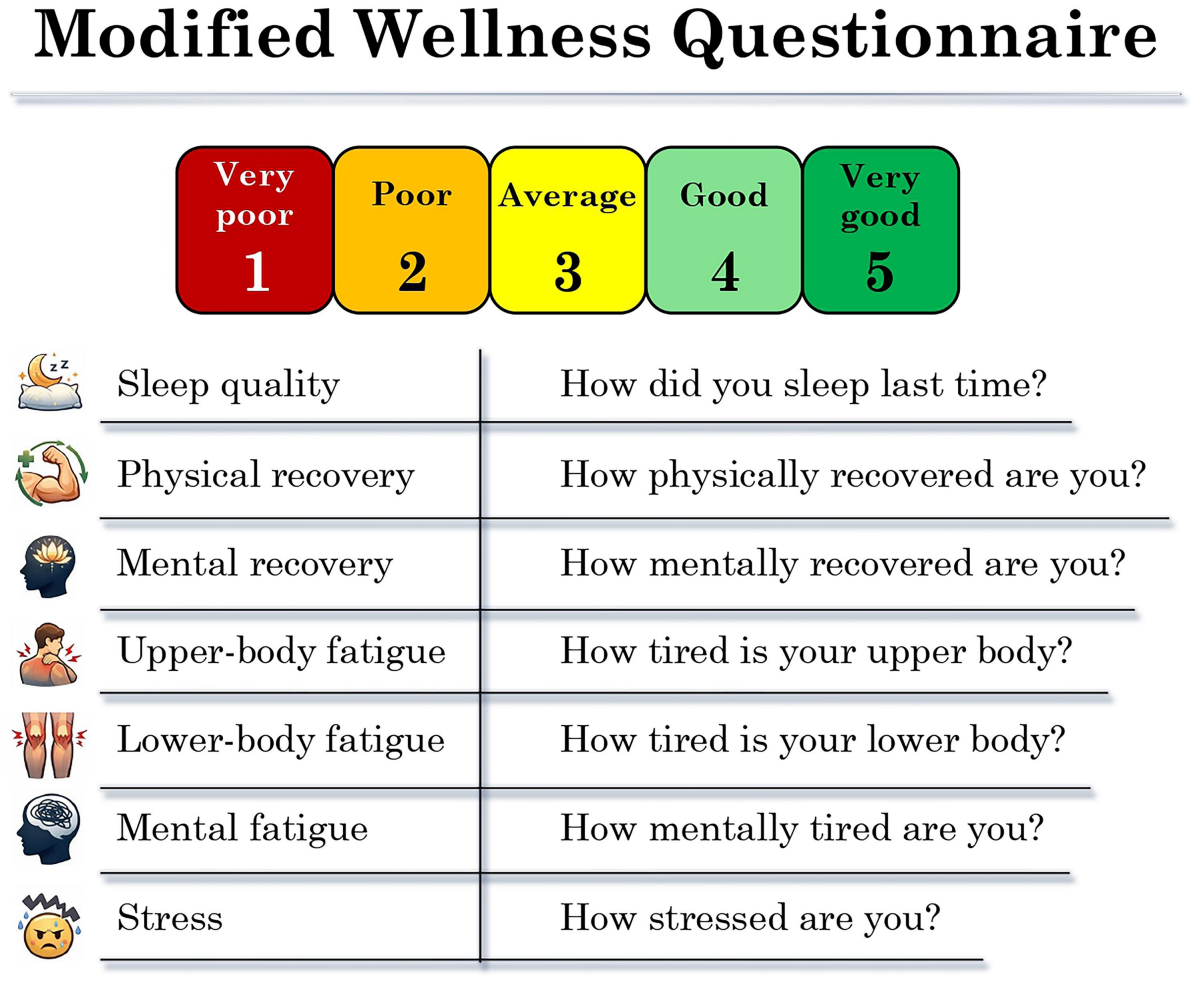
Item structure and scoring logic of the modified wellness questionnaire. Higher scores indicate a more favorable wellness status for all items; therefore, higher values on the stress and fatigue items reflect lower perceived stress and lower perceived fatigue. Composite recovery and fatigue scores were calculated as the mean of their component items.

Perceived wellness was assessed using four monitored domains: sleep quality, recovery, fatigue, and stress (Figure 1). Recovery was represented by two items assessing physical and mental recovery, while fatigue was represented by three items (upper-body fatigue, lower-body fatigue, mental fatigue). Sleep quality and stress were assessed as single-item indicators. All items were rated on a 5-point Likert scale ranging from 1 (“very poor”) to 5 (“very good”). Scores were coded so that higher values reflected a more favorable wellness status; therefore, for stress- and fatigue-related items, higher scores indicated lower perceived stress or fatigue. To facilitate interpretation in the applied setting, all items were scored in the same direction. Although this scoring approach simplified applied interpretation by aligning all items in the same direction, it may limit direct comparability with questionnaires in which higher fatigue or stress scores indicate greater symptom severity. Accordingly, higher values on the fatigue-related and stress-related items indicate lower perceived fatigue and lower perceived stress, respectively. For multi-item domains, mean scores were calculated to create composite recovery and fatigue scores. These composite scores were intended to provide parsimonious indicators of the broader recovery and fatigue domains for practical monitoring purposes. For descriptive purposes, wellness scores were summarized across measurement occasions, whereas all reliability and mixed-effects analyses were conducted on the full repeated-measures dataset. The questionnaire was administered on each measurement day under the same team monitoring routine. Athletes completed the questionnaire through the TalentX application after receiving a standardized electronic notification before training. The same digital response format was used throughout the monitoring period. Completion was supervised by the sport science staff to support compliance, while responses remained self-reported by the athletes.

### 2.6 External monitoring variables

Nocturnal sleep duration and heart rate variability (HRV) were recorded during sleep using a wrist-worn WHOOP device (WHOOP Inc., Boston, MA, USA). WHOOP monitoring began after the first two questionnaire assessment occasions and included 17 players. Sleep duration and HRV were obtained from the WHOOP platform, which uses proprietary algorithms to process actigraphy and photoplethysmography signals (21, 22). No additional thresholds for minimum wear time or signal quality were defined. For each analysis, only observations with an available value for the relevant variable were included; missing values were not imputed. A previous validation study of WHOOP devices showed high agreement with ECG-derived RMSSD, with an intraclass correlation of 0.99, mean bias of −4.5 ± 3.9 ms, and limits of agreement of ± 7.6 ms (21). Earlier WHOOP validation work also reported acceptable agreement for heart rate, although HRV indices such as lnRMSSD required more cautious interpretation because limits of agreement approached or exceeded the smallest worthwhile change and coefficient of variation (22). Therefore, the present WHOOP-derived sleep and HRV values were interpreted as field-monitoring estimates rather than laboratory-grade measurements.

Training session RPE was obtained approximately 10 min after each training session using the Borg CR-10 scale. The session-RPE approach originally proposed by Foster et al. (2001) was used to derive internal training load from perceived exertion and session duration (23). When more than one training session occurred on the same day, s-RPE was calculated separately for each session as RPE multiplied by session duration in minutes, and daily s-RPE was calculated as the sum of s-RPE values for that player and day. Importantly, the s-RPE method has also been examined in water polo, showing strong associations with HR-based internal load estimates in youth male water polo players (24).

### 2.7 Statistical analysis

Statistical analyses were conducted using JASP software (version 0.95.4, JASP Team, Amsterdam, Netherlands). The analyses were selected to describe internal measurement characteristics of the questionnaire during repeated field use. Descriptive statistics (means, standard deviations, and modes) were calculated for all questionnaire items and composite scores. Distributional properties were examined using skewness and kurtosis values. Item-level relationships were examined using Spearman correlation coefficients. As these correlations were calculated on pooled repeated-measures data, they were interpreted descriptively and not as independent between-athlete associations. The magnitude of the correlation was determined by Hopkins et al. (2009): trivial (< 0.1), weak (0.1–0.3), moderate (0.3–0.5), strong (0.5–0.7), very strong (0.7–0.9), and near perfect (> 0.9) (25). Internal consistency was assessed only for multi-item composites using Cronbach's alpha and, where applicable, McDonald's omega coefficients. Because the recovery composite consisted of only two items, McDonald's omega was reported only for the fatigue composite. Values of ≥ 0.70 were considered acceptable. Intraclass correlation coefficients were calculated to estimate between-athlete reliability across repeated measurement occasions. ICC (2,1), a two-way random-effects model for absolute agreement based on single measurements, was selected for the items and composite scores. For each composite score, standard error of measurement (SEM) was calculated from the standard deviation (SD) as SD×√(1−ICC) and minimal detectable change at the 95% confidence level (MDC_95_) as SEM×1.96×√2. Within-questionnaire associations were examined using linear mixed-effects models predicting the reverse-coded stress score from recovery and fatigue composite scores. Models were fitted with a random intercept for Player ID to account for repeated observations within players, using restricted maximum likelihood estimation and Satterthwaite approximation for degrees of freedom. Recovery and fatigue were first examined in separate models and then simultaneously. A sensitivity model additionally included measurement occasion as a fixed effect. Variance inflation factors were calculated to assess multicollinearity between recovery and fatigue. Exploratory external association analyses were conducted using separate linear mixed-effects models to examine whether sleep duration predicted sleep quality and recovery, and whether HRV predicted the reverse-coded stress score. Daily s-RPE was also inspected in relation to wellness scores using Spearman correlations and additional mixed-effects models. Potential delayed associations were examined using two additional lagged mixed-effects models. Previous day daily s-RPE was entered as a predictor of next-day recovery and fatigue, with the corresponding previous day wellness score included as a covariate, and Player ID as a random intercept. Only consecutive day pairs were retained. The level of statistical significance was set at *p* < 0.05 for all analyses. No adjustment was made for multiple comparisons.

## 3 Results

The monitoring protocol comprised 12 repeated administrations per athlete, yielding 216 planned and completed observations; no included participant was lost to follow-up.

Inter-item associations are presented descriptively to summarize the internal patterning of the questionnaire across the repeated-measures dataset (Table 1).

**Table 1:** Inter-item correlations among the modified wellness questionnaire items.

Variables	Sleep quality	Physical recovery	Mental recovery	Upper-body fatigue	Lower-body fatigue	Mental fatigue	Stress
Sleep quality	-	0.43	0.33	0.34	0.31	0.25	0.25
Physical recovery	0.43	-	0.51	0.73	0.68	0.46	0.30
Mental recovery	0.33	0.51	-	0.50	0.39	0.65	0.24
Upper-body fatigue	0.34	0.73	0.50	-	0.67	0.56	0.21
Lower-body fatigue	0.31	0.68	0.39	0.67	-	0.49	0.24
Mental fatigue	0.25	0.46	0.65	0.56	0.49	-	0.20
Stress	0.25	0.30	0.24	0.21	0.24	0.20	-

Spearman correlations were computed on the pooled repeated-measures dataset. Higher scores indicate a more favorable status for all items.

Between-athlete reliability estimates are presented in Table 2. Recovery-related items showed low intraclass correlation coefficient (ICC) values. Sleep quality showed a low ICC [ICC(2,1) = 0.09, 95% CI 0.02–0.24], as did physical recovery [ICC(2,1) = 0.13, 95% CI 0.05–0.30] and mental recovery [ICC(2,1) = 0.04, 95% CI −0.01–0.17]. Mental fatigue exhibited slightly higher reliability compared with recovery-related items [ICC(2,1) = 0.11, 95% CI 0.03–0.28]. In contrast, perceived stress demonstrated moderate between-athlete reliability [ICC(2,1) = 0.32, 95% CI 0.18–0.55]. Upper- and lower-body fatigue showed moderate ICC values, with upper-body fatigue yielding an ICC(2,1) of 0.31 (95% CI 0.17–0.53) and lower-body fatigue demonstrating the highest reliability among fatigue-related items [ICC(2,1) = 0.35, 95% CI 0.20–0.57].

**Table 2:** Descriptive statistics and between-athlete reliability of the modified wellness questionnaire items.

Variables	Mean	SD	Mode	ICC (2,1)	95% CI
Sleep quality	3.54	0.83	4	0.09	0.02–0.24
Physical recovery	3.32	0.81	3	0.13	0.05–0.30
Mental recovery	3.81	0.64	4	0.04	−0.01–0.17
Upper-body fatigue	3.39	0.91	4	0.31	0.17–0.53
Lower-body fatigue	3.31	0.89	3	0.35	0.20–0.57
Mental fatigue	3.85	0.68	4	0.11	0.03–0.28
Stress	4.09	0.81	4	0.32	0.18–0.55

ICC values represent between-athlete reliability across repeated assessments. Higher scores on all items indicate a more favorable status.

The recovery and fatigue composites showed ICC (2,1) values of 0.09 (95% CI 0.02–0.23) and 0.31 (95% CI 0.17–0.53), respectively. The SEM was relatively high for both composites (SEM = 0.61 for recovery and 0.59 for fatigue), resulting in large minimal detectable changes (MDC_95_ = 1.69 and 1.64, respectively). The fatigue composite demonstrated good internal consistency (*α* = 0.81, 95% CI 0.71–0.90; McDonald's *ω* = 0.83, 95% CI 0.79–0.87). The recovery composite showed acceptable internal consistency (*α* = 0.70).

Linear mixed-effects models predicting the reverse-coded stress score are presented in Table 3. Recovery was positively associated with the stress score when entered alone and remained significant when fatigue was included in the model. Fatigue was significant only when entered as a single predictor and was no longer significant in the combined model. In the sensitivity model including measurement occasion, recovery remained significant, whereas fatigue and session (measurement occasion) were not significant. Collinearity diagnostics indicated no multicollinearity between recovery and fatigue (VIF = 2.61, tolerance = 0.38).

**Table 3:** Linear mixed-effects models predicting the reverse-coded stress score.

Models	Predictor	Estimate	SE	t	p
Model 1	Recovery	0.315	0.074	4.263	< 0.001
Model 2	Fatigue	0.219	0.076	2.886	0.004
Model 3	Recovery	0.370	0.118	3.130	0.002
	Fatigue	−0.071	0.118	−0.595	0.552
Model 4	Recovery	0.366	0.118	3.101	0.002
	Fatigue	−0.084	0.119	−0.709	0.479
	Session	−0.017	0.013	−1.287	0.200

All models included a random intercept for Player ID. Higher stress scores indicate lower perceived stress.

WHOOP-derived sleep duration and HRV data were available for 125 observations from 17 players, while daily s-RPE data were available for 186 observations from all 18 players. Sleep duration was positively associated with perceived sleep quality and recovery. HRV was positively associated with the reverse-coded stress score. Daily s-RPE was not meaningfully associated with concurrent wellness scores in either exploratory correlation analyses or additional mixed-effects models (Table 4).

**Table 4:** Exploratory external association mixed-effects models for selected wellness outcomes.

Outcome	External predictor	Estimate	SE	F	df	p
Sleep quality	Sleep duration (h)	0.419	0.080	27.54	1, 121.14	< 0.001
Recovery	Sleep duration (h)	0.166	0.065	6.467	1, 117.70	0.012
Stress	HRV	0.010	0.003	10.27	1, 47.40	0.002

HRV, heart rate variability.

All models included a random intercept for Player ID and used Satterthwaite approximation for degrees of freedom. Higher scores indicate a more favorable status for all questionnaire outcomes.

Previous day s-RPE was not associated with next-day recovery after adjustment for previous day recovery [estimate = −0.000155 per AU, SE = 0.000095, t(134.03) = −1.632, *p* = 0.105]. Higher previous day s-RPE was, however, associated with a lower next-day fatigue score after adjustment for previous day fatigue [estimate = −0.000186 per AU, SE = 0.000085, t(130.65) = −2.188, *p* = 0.030].

## 4 Discussion

The present study provides a preliminary field-based description of selected internal measurement characteristics of a modified wellness questionnaire in an elite male national water polo team. The findings describe how the questionnaire behaved internally during short-term repeated use in its intended applied setting; however, the findings should not be interpreted as full psychometric validation.

Intraclass correlation analyses revealed generally low between-athlete reliability across most wellness items, particularly those related to recovery and sleep. In the present study, ICC values were interpreted as indicators of between-athlete reliability across repeated assessments, not as direct evidence of test-retest reliability or responsiveness. Low ICC values may reflect within-athlete variability over time, but they do not by themselves demonstrate responsiveness to meaningful training-related changes. This pattern suggests that the scores varied more across repeated occasions within players than between players (26). These low ICC values limit the use of the questionnaire for between-athlete comparisons. At the same time, in a short-term monitoring context, they may also reflect within-athlete variability, which is relevant for state-like wellness indicators. Additionally, subjective wellness measures are explicitly intended to capture short-term, state-like changes in response to training load, recovery status and psychosocial stressors (2, 18). A similar pattern of pronounced within-athlete variability has been reported in previous monitoring studies, where subjective wellness measures primarily reflected short-term state fluctuations rather than stable individual characteristics (27). The results should therefore be viewed as an initial evaluation in a specific elite water polo setting.

Recovery-related items, including sleep quality and physical and mental recovery, exhibited the lowest ICC values, suggesting pronounced day-to-day variability. This finding aligns with previous research demonstrating that subjective recovery and wellness measures are highly sensitive to acute training and everyday life stressors in elite athletes (1, 2). In contrast, perceived stress and physical fatigue items demonstrated moderate between-athlete reliability, indicating a relatively more stable individual component alongside meaningful within-athlete variation. Similar patterns have been reported in monitoring studies distinguishing between trait- and state-like components of athlete wellness (18, 20).

However, the present findings should not be interpreted as evidence of full instrument validation. In particular, the study did not examine structural validity, cross-context stability, responsiveness, or criterion validity against performance, injury, or health outcomes. The external association analyses provide only exploratory information on selected relationships, and the results are therefore best understood as preliminary measurement-property evidence in a specific elite water polo setting.

Internal consistency analyses provided preliminary information on the coherence of the predefined recovery and fatigue item groups. The fatigue composite demonstrated good internal consistency, while the recovery composite showed acceptable internal consistency based on Cronbach's alpha. However, these findings do not confirm dimensionality, because structural validity was not tested. The recovery and fatigue scores should therefore be interpreted as theoretically and practically defined composite indicators rather than empirically confirmed latent dimensions. The present findings therefore suggest that the recovery and fatigue composite scores were related but not redundant within this dataset, which is compatible with conceptual distinctions between recovery and fatigue in athlete monitoring (18). In addition, the relatively large MDC_95_ values suggest that small day-to-day score changes should be interpreted cautiously at the individual level, whereas larger deviations may warrant closer contextual interpretation in applied monitoring. Because athlete stability was not established and wellness may have changed from day to day during the training camp, the SEM and MDC_95_ values may reflect both real changes and measurement error.

Because recovery, fatigue, and stress were all derived from the same questionnaire, the mixed-model analyses should be interpreted as evidence of theoretically coherent internal associations rather than as a definitive test of construct validity. In this respect, the analyses describe the internal coherence among questionnaire domains, while leaving broader validity questions open for future studies. Recovery remained associated with the stress score when fatigue was included in the same model. Given the coding of the stress item, this indicates that higher perceived recovery was associated with lower perceived stress within the questionnaire data. However, because all variables were derived from the same self-report instrument, this association should be interpreted as internal coherence among theoretically related domains rather than as evidence of causal direction, construct validation, or an independent psychological mechanism. More broadly, this pattern is compatible with previous research emphasizing the relevance of recovery in the relationship between training demands, psychological strain, and perceived wellness, and with studies showing that subjective recovery measures may help explain athletes' perceived responses to training (2, 18, 28, 29).

The association between sleep duration and perceived sleep quality was consistent with the expected relationship between an external sleep quantity marker and the corresponding questionnaire item. The association between sleep duration and recovery also suggests that recovery scores were partly related to sleep-related information during the training camp. In addition, higher HRV was associated with a more favorable stress score, which is directionally consistent with the interpretation of lower perceived stress alongside more favorable autonomic status. However, these findings should be interpreted cautiously because external data were incomplete, the analyses were exploratory, and the models do not establish criterion validity or responsiveness. Because wellness was assessed before training, whereas daily s-RPE represents the load accumulated during that day, the absence of concurrent associations is not unexpected. The lagged finding suggests that perceived fatigue may reflect previous day training load more clearly than same day load. However, this pattern was not observed for recovery and should be considered preliminary given the small effect and short monitoring period.

## 5 Limitations

Several limitations must be addressed. The sample was small, male only, and drawn from a single national-level water polo team. No sample size calculation was performed for the measurement properties examined. The analytical sample included all eligible players who completed the full two-week monitoring period. These limit generalizability to other teams, female athletes, youth or sub-elite populations, and different competitive contexts. Wellness responses and self-assessment processes may differ across age groups, sex, training history, and competitive level; therefore, the findings should not be extrapolated beyond the examined adult elite male sample. The monitoring period covered a short timeframe of two weeks within an intensive national team training camp. Although this duration was suitable for capturing observed within-athlete variability, it may not reflect long-term adaptations or seasonal changes in perceived wellness. Accordingly, the present findings should be interpreted as sample-specific preliminary evidence rather than definitive psychometric confirmation of the instrument. An additional limitation is that the adaptation process was not accompanied by formal cognitive interviewing or an external content-validity panel. Therefore, the adaptation should be considered pragmatic and context-specific rather than a full content-validity evaluation. Moreover, the present sample was not designed to support factor-analytic testing; therefore, the structural validity remains to be examined in a larger and more heterogeneous cohort. Although the repeated-measures design increased the number of observations, these observations were nested within a small number of independent athletes and therefore cannot substitute for a larger independent sample required for factor analysis. The requirement to complete the full monitoring period may have introduced selection bias. Information bias and residual confounding also cannot be excluded because questionnaire scores were self-reported, WHOOP data were processed using proprietary algorithms, and not all factors influencing daily wellness were included in the models. Lastly, the external association analyses were limited to sleep duration, HRV and s-RPE.

## 6 Future applications

If used in applied practice, the modified wellness questionnaire should be interpreted as a descriptive adjunct to existing athlete monitoring routines (1). The present findings suggest that the questionnaire may help practitioners summarize short-term within-athlete patterns in perceived wellness during intensive national team training periods. The exploratory external associations with sleep duration and HRV support the value of interpreting questionnaire scores alongside complementary sleep and physiological monitoring data. At the same time, the findings do not support using the questionnaire as a direct substitute for training load monitoring. Interpretation should focus on individual response profiles and deviations from personal baselines rather than comparisons between athletes. The questionnaire should not be used as a stand-alone tool for diagnosing fatigue or recovery status, quantifying training adaptation, or prescribing training modifications. Any practical use should be combined with contextual information, including training load data, sleep information, health-related observations by the medical staff, performance observations, and practitioners' judgment. Until broader external validity and responsiveness are examined, the questionnaire should be regarded as a descriptive monitoring aid.

## 7 Conclusion

In an elite national team water polo environment, the modified wellness questionnaire showed interpretable short-term patterns in perceived wellness. Exploratory associations with sleep duration and HRV were observed for selected domains. Concurrent s-RPE was not clearly related to wellness scores, although a small lagged association was observed with next day fatigue. The questionnaire may be useful as a descriptive monitoring aid, but further validation is needed before it can be used as a stand-alone decision-making tool.

## Data Availability

The raw data supporting the conclusions of this article will be made available by the authors, without undue reservation.
